# A Case Report of Cardiac Amyloidosis Highlighting the Importance of Strain Analysis

**DOI:** 10.1155/2021/5673364

**Published:** 2021-10-12

**Authors:** Andres Cordova Sanchez, Ryan Murphy, Suman Rao, Fidel Martinez, Stephanie Bryant, Debanik Chaudhuri

**Affiliations:** ^1^Department of Medicine, SUNY Upstate Medical University, Syracuse, NY 13210, USA; ^2^College of Medicine, SUNY Upstate Medical University, Syracuse, NY 13210, USA; ^3^Division of Cardiology, SUNY Upstate Medical University, Syracuse, NY 13210, USA; ^4^Department of Pathology, SUNY Upstate Medical University, Syracuse, NY 13210, USA

## Abstract

Cardiac involvement in light-chain (AL) amyloidosis has a high mortality. Once cardiac symptoms are present, it is important to make a diagnosis as there is an inverse relationship between mortality and time of diagnosis. Echocardiography is usually one of the first tests performed. But strain analysis, which can provide important clues, is not routinely performed. This is a case of AL amyloidosis presenting with heart failure in which echocardiographic strain analysis was vital for its diagnosis.

## 1. Introduction

Amyloidosis consists of the accumulation of misfolded beta-pleated proteins anywhere in the body leading to organ dysfunction. Primary amyloidosis or AL amyloidosis is the result of a plasma cell dyscrasia that produces kappa or lambda monoclonal light chains which are deposited as amyloid fibrils in the body [1].

AL amyloidosis is the most common type of amyloid. It represents approximately half of the diagnosed amyloid disorders. In 2015, the prevalence of AL amyloidosis had more than doubled compared to 2007, with a prevalence of 50.1 cases per million in 2015, with a somewhat similar incidence [2]. Due to chemotherapy advances, survival of AL amyloidosis has increased drastically from 18 months before 2005 to more than 5 years [3].

Symptomatology depends on the affected organ. The most common associated conditions are nephrotic syndrome, heart failure, and peripheral neuropathy. This wide variety of nonspecific symptoms make the diagnosis of AL amyloidosis challenging. Patients are usually diagnosed once severe organ damage is present [4].

Due to the frequent cardiac involvement seen with AL amyloidosis, an echocardiogram is likely to be part of the initial workup. Missing the signs of amyloidosis in this test can result in poor outcomes. Early diagnosis is of vital importance given the inverse relationship between time after the onset of cardiac symptoms and survival [5].

Here, we present a case of a patient who had approximately 4 months of heart failure with preserved ejection fraction and years of polyneuropathy in whom the echocardiogram findings during strain analysis were vital in identifying amyloidosis. Highlighting the importance of cardiac imaging in its diagnosis. The purpose of this case report is to raise awareness about the importance of strain analysis in the early recognition of AL amyloidosis.

## 2. Case Report

A 72-year-old male is with a past medical history of pulmonary embolism, factor V Leiden deficiency, atrial fibrillation, and a 9-year history of unspecified/undiagnosed muscle weakness and pain of the lower extremities. For 4 months, our patient experienced progressive dyspnea, generalized edema, bilateral pleural effusions, and recurrent syncope that was diagnosed as heart failure with preserved ejection fraction.

**Figure 1:**
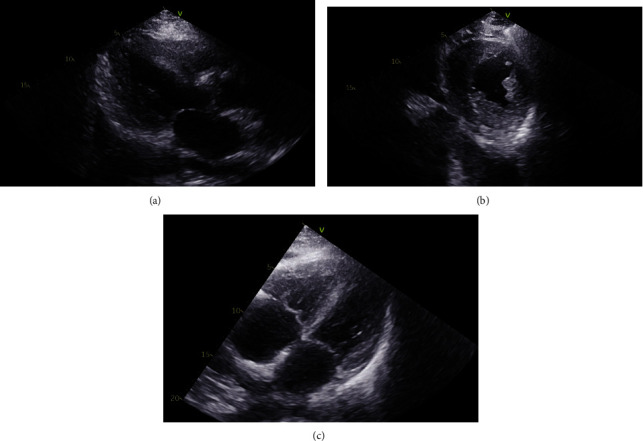
Previous echocardiogram (a). Parasternal long axis (b). Parasternal short axis (c). Apical four-chamber showing moderate concentric left ventricular hypertrophy, right ventricular dilation, and moderately dilated atria.

Previous echocardiograms (Figure 1) had shown moderate concentric left ventricular hypertrophy, normal ejection fraction, hypokinesis of basoseptal left ventricular wall, with right ventricular dilation, moderately dilated atria, and pulmonary hypertension at around 40 mmHg; tissue Doppler was not performed. Our patient presented with monthly episodes of dyspnea and syncope which were managed as heart failure exacerbation.

During this admission, he came after another syncopal episode which happened while walking associated with presyncopal nausea, dizziness, and a sensation of heat all over the body. He had a witnessed loss of consciousness of 2 minutes, with no postsyncopal symptoms.

On arrival, he was hemodynamically stable, his laboratory results were significant for a pro-BNP of 13,458 and high sensitivity troponin T of 0.16, the electrocardiogram showed atrial fibrillation with a ventricular rate of 90 beats per minute, and a chest X-ray shows bilateral pleural effusion.

Cardiac catheterization showed normal left ventricular systolic function with an ejection fraction of 55%, without wall motion abnormalities, elevated left ventricular end-diastolic pressure at 25 mmHg, elevated mean pulmonary artery pressure of 37 mmHg, and a capillary wedge pressure of 30 mmHg. There was no significant coronary artery disease.

**Figure 2:**
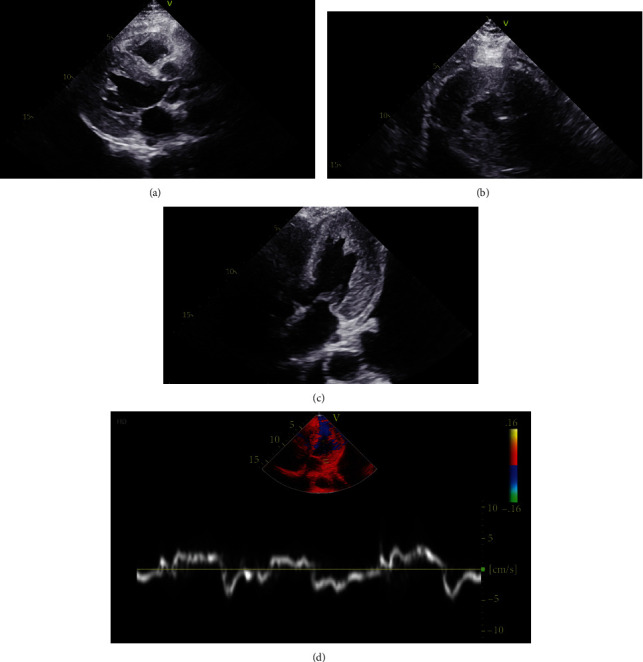
Echocardiogram (a). Parasternal long axis (b). Parasternal short axis (c). Apical two-chamber of left ventricle and (d) tissue Doppler showing severe left ventricular hypertrophy and diastolic dysfunction.

**Figure 3:**
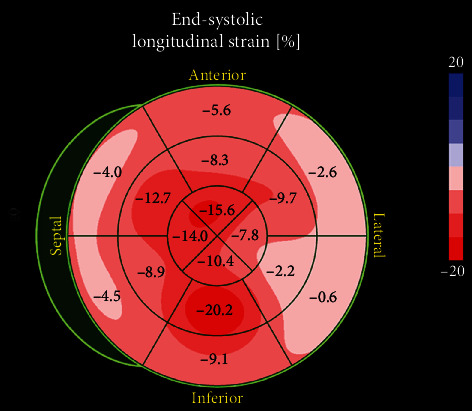
Echocardiographic strain analysis showing a relative sparing of the apex compared to the bases.

An echocardiogram was repeated, this time with tissue Doppler and strain analysis (Figures 2 and 3) which was significant for a cherry on top appearance with relative basal sparing, increased *E*/*e*′ ratio at 23.6, decreased *e*′ at 2 cm/s, severe concentric hypertrophy, and an ejection fraction of 37%.

**Figure 4:**
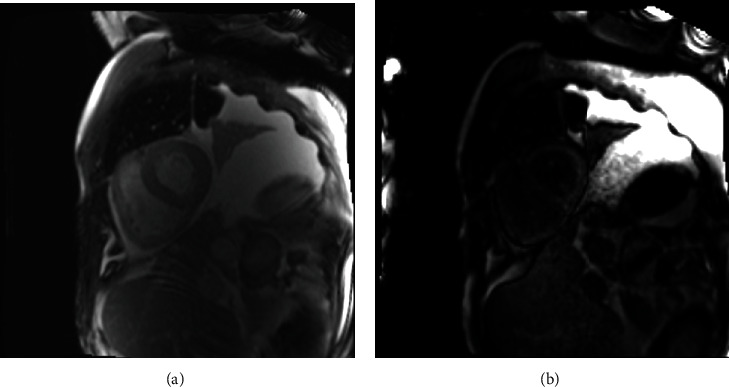
Cardiac magnetic resonance; (a) left ventricle hypertrophy, (b) abnormal nulling of the myocardium.

Cardiac magnetic resonance imaging confirmed an ejection fraction of 37% with diffuse hypokinesis of both ventricles, diffusely thickened left ventricle, and abnormal nulling of the myocardium (Figure 4).

**Figure 5:**
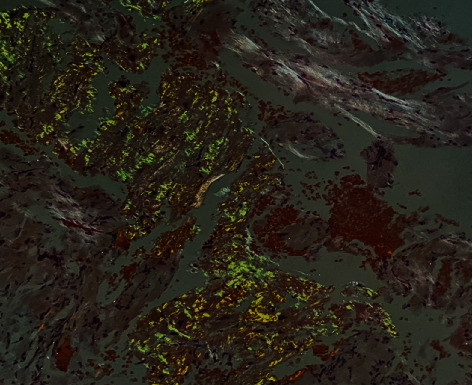
Cardiac biopsy with Congo red stain, demonstrating apple green birefringence in polarized light, consistent with amyloidosis.

Serum and urine electrophoresis did not show an M spike, and no paraproteins were found in immunofixation. However, increased kappa and lambda free light chains with a decreased kappa/lambda ratio were found on serum-free immunoglobulin analysis. Subsequent bone marrow biopsy showed 4.2% clonal plasma cells. Fat pad and two bone marrow biopsies were negative for Congo red staining. Finally, a cardiac biopsy was positive for amyloid with Congo red staining (Figure 5) and demonstrated AL amyloid on mass spectrometry.

Chemotherapy with daratumumab, bortezomib, cyclophosphamide, and dexamethasone was started, and normalization of kappa and lambda chain levels was achieved after the second cycle.

## 3. Discussion

Cardiac involvement in AL amyloidosis presents with arrhythmias and/or rapidly progressing heart failure. Amyloid fibrils cause stiffening of the heart which explains restrictive physiology found on the echocardiogram. Once a patient develops cardiac symptoms, they usually have a large amount of amyloid in the heart [6]. Amyloid deposit in the atrium causes an irregular surface that makes these patients prone to thrombus formation, irrespective of atrial fibrillation development [6].

Oubari et al. found a significant inverse relationship between time of diagnosis after the onset of cardiac symptoms and 5-year survival. Survival ranged from 79% if diagnosed during the first 6 months to 19% after 19 months [5]. This difference was statistically significant when using a 1-year cutoff, indicating there is a 1-year window for diagnosis after the onset of cardiac symptoms. Heart function in patients with AL amyloidosis does not appear to recover after treatment [7]. The main cause for delayed diagnosis is insufficient physician awareness [5].

Initial workup for AL amyloidosis includes immunofixation, serum and urine electrophoresis, and immunoglobulin free light chains assay. Serum and urine protein electrophoresis can be negative in half of the patients. Immunofixation and serum-free light chain are more sensitive tests. Serum-free light chain immunoglobulin can be seen in >90% of the cases. However, it is important to remember that a negative immunofixation does not rule out the disease [6].

Definitive diagnosis requires identification of amyloid with Congo red staining and identification of the type of misfolded protein [3, 8]. Specific organ biopsy is required only 15% of the time [3]. Fat pad and bone marrow biopsy are diagnostic 67% and 72% of the time, respectively. Cardiac and renal biopsies are positive 100% of the time [9]. In our case, fat pad and two different bone marrow biopsies were negative. However, due to high suspicion, we confirmed the diagnosis of AL amyloidosis with endomyocardial biopsy.

Due to the frequent cardiac involvement, the first signs suggestive of AL amyloidosis will often be found during cardiac workup. Pro-BNP and troponin levels are typically elevated [6]; classically low amplitude QRS is found on electrocardiogram [10].

Echocardiogram will likely be part of the work-up ordered early in the course of the disease. Accurately identifying the echocardiographic signs of amyloidosis can expedite diagnosis and increase life expectancy. Severe ventricular wall thickening, heart failure with preserved ejection fraction, and restrictive pattern on Doppler are typical signs seen in cardiac amyloidosis. In tissue Doppler, *E*/*e*′ can be >15 due to the elevated filling pressures; transmitral A wave can be absent with atrial involvement [6]. Strain analysis showing relative apical sparing with a “cherry on top” appearance seems to be the most sensitive and specific finding of amyloidosis [11].

Cardiac magnetic resonance is another important diagnostic tool; classic signs of amyloidosis are difficult nulling of the myocardium after gadolinium administration and late gadolinium enhancement in ventricles and atria [6]. In AL amyloidosis, this delayed enhancement is typically global, subendocardial, and does not follow a specific arterial distribution [12].

^99m^Tectetium pyrophosphate uptake is seen with TTR amyloidosis but not with AL amyloid and can help differentiate between these two [6] but is not a test used when there is high suspicion for AL amyloidosis.

Management of AL amyloidosis consists of medical optimization of the organs involved and chemotherapy to eliminate the plasma cells responsible for the abnormal free light chain in the body.

Our patient had sudden onset, rapidly progressive heart failure, associated with syncope, difficult to control atrial fibrillation, and a long history of undiagnosed lower extremity pain and weakness. Initially, the suspicion for cardiac amyloidosis was low. However, once severe left ventricular hypertrophy, biatrial enlargement, restrictive physiology, and global longitudinal strain with relative apical sparing were found on echocardiogram, our suspicion for it was very high. We consider that his history of undiagnosed muscular weakness was a peripheral neuropathy and was part of the systemic involvement of AL amyloidosis. The diagnosis was confirmed with an endomyocardial biopsy, and chemotherapy was started.

## 4. Conclusion

This case highlights the importance of echocardiography in the diagnosis of AL amyloidosis. The characteristic result from strain analysis was strongly indicative of cardiac amyloidosis. We believe that tissue Doppler with strain should be included in all patients with heart failure as it has the potential to provide vital information to perform an accurate diagnosis.

## Data Availability

No data were used to support this study.
